# Toward Precision Medicine in ADHD

**DOI:** 10.3389/fnbeh.2022.900981

**Published:** 2022-07-06

**Authors:** Jan Buitelaar, Sven Bölte, Daniel Brandeis, Arthur Caye, Nina Christmann, Samuele Cortese, David Coghill, Stephen V. Faraone, Barbara Franke, Markus Gleitz, Corina U. Greven, Sandra Kooij, Douglas Teixeira Leffa, Nanda Rommelse, Jeffrey H. Newcorn, Guilherme V. Polanczyk, Luis Augusto Rohde, Emily Simonoff, Mark Stein, Benedetto Vitiello, Yanki Yazgan, Michael Roesler, Manfred Doepfner, Tobias Banaschewski

**Affiliations:** ^1^Department of Cognitive Neuroscience, Donders Institute for Brain, Cognition and Behaviour, Radboud University Medical Centre, Nijmegen, Netherlands; ^2^Karakter Child and Adolescent Psychiatry University Center, Nijmegen, Netherlands; ^3^Center of Neurodevelopmental Disorders (KIND), Centre for Psychiatry Research, Department of Women's and Children's Health, Karolinska Institutet, Solna, Sweden; ^4^Child and Adolescent Psychiatry, Stockholm Health Care Services, Stockholm, Sweden; ^5^Curtin Autism Research Group, School of Occupational Therapy, Social Work and Speech Pathology, Curtin University, Perth, WA, Australia; ^6^Department of Child and Adolescent Psychiatry and Psychotherapy, Central Institute of Mental Health, Medical Faculty Mannheim/Heidelberg University, Mannheim, Germany; ^7^Department of Child and Adolescent Psychiatry and Psychotherapy, Psychiatric Hospital, University of Zurich, Zurich, Switzerland; ^8^Department of Psychiatry, Hospital de Clinicas de Porto Alegre, Federal University of Rio Grande do Sul, Porto Alegre, Brazil; ^9^National Institute of Developmental Psychiatry for Children and Adolescents, São Paulo, Brazil; ^10^Centre for Innovation in Mental Health, Academic Unit of Psychology, Faculty of Environmental and Life Sciences, University of Southampton, Southampton, United Kingdom; ^11^Clinical and Experimental Sciences (CNS and Psychiatry), Faculty of Medicine, University of Southampton, Southampton, United Kingdom; ^12^Solent National Health System Trust, Southampton, United Kingdom; ^13^Hassenfeld Children's Hospital at NYU Langone, New York University Child Study Center, New York, NY, United States; ^14^Division of Psychiatry and Applied Psychology, School of Medicine, University of Nottingham, Nottingham, United Kingdom; ^15^Departments of Paediatrics and Psychiatry, Royal Children's Hospital, University of Melbourne, Melbourne, VIC, Australia; ^16^Departments of Psychiatry, Neuroscience and Physiology, SUNY Upstate Medical University, Syracuse, New York, NY, United States; ^17^Departments of Human Genetics and Psychiatry, Donders Institute for Brain, Cognition and Behaviour, Radboud University Medical Center, Nijmegen, Netherlands; ^18^Medice Arzneimittel Pütter GmbH & Co. KG, Iserlohn, Germany; ^19^Department of Cognitive Neuroscience, Donders Institute for Brain, Cognition and Behaviour, Radboud University Medical Center, Nijmegen, Netherlands; ^20^King's College London, Social, Genetic and Developmental Psychiatry Centre, Institute of Psychiatry, Psychology and Neuroscience, London, United Kingdom; ^21^Amsterdam University Medical Center, Location VUMc, Amsterdam, Netherlands; ^22^PsyQ, Expertise Center Adult ADHD, The Hague, Netherlands; ^23^Department of Psychiatry, Donders Institute for Brain, Cognition and Behaviour, Radboud University Medical Center, Nijmegen, Netherlands; ^24^Department of Psychiatry, Icahn School of Medicine at Mount Sinai, New York, NY, United States; ^25^Department of Psychiatry, Faculdade de Medicina FMUSP, Universidade de São Paulo, São Paulo, Brazil; ^26^ADHD Outpatient Program and Developmental Psychiatry Program, Hospital de Clinica de Porto Alegre, Federal University of Rio Grande do Sul, Porto Alegre, Brazil; ^27^Department of Child and Adolescent Psychiatry, Institute of Psychiatry, Psychology and Neuroscience, King's College London, United Kingdom; ^28^Department of Psychiatry and Behavioral Sciences, Seattle, WA, United States; ^29^Department of Public Health and Pediatric Sciences, Section of Child and Adolescent Neuropsychiatry, University of Turin, Turin, Italy; ^30^Department of Public Health, Johns Hopkins University, Baltimore, MA, United States; ^31^GuzelGunler Clinic, Istanbul, Turkey; ^32^Yale Child Study Center, New Haven, CT, United States; ^33^Institute for Forensic Psychology and Psychiatry, Neurocenter, Saarland, Germany; ^34^Department of Child and Adolescent Psychiatry, Psychosomatics and Psychotherapy, Medical Faculty of the University of Cologne, Cologne, Germany; ^35^School for Child and Adolescent Cognitive Behavioural Therapy, University Hospital of Cologne, Cologne, Germany

**Keywords:** Attention-Deficit Hyperactivity Disorder (ADHD), precision medicine, biomarker, heterogeneity, inter-individual variability

## Abstract

Attention-Deficit Hyperactivity Disorder (ADHD) is a complex and heterogeneous neurodevelopmental condition for which curative treatments are lacking. Whilst pharmacological treatments are generally effective and safe, there is considerable inter-individual variability among patients regarding treatment response, required dose, and tolerability. Many of the non-pharmacological treatments, which are preferred to drug-treatment by some patients, either lack efficacy for core symptoms or are associated with small effect sizes. No evidence-based decision tools are currently available to allocate pharmacological or psychosocial treatments based on the patient's clinical, environmental, cognitive, genetic, or biological characteristics. We systematically reviewed potential biomarkers that may help in diagnosing ADHD and/or stratifying ADHD into more homogeneous subgroups and/or predict clinical course, treatment response, and long-term outcome across the lifespan. Most work involved exploratory studies with cognitive, actigraphic and EEG diagnostic markers to predict ADHD, along with relatively few studies exploring markers to subtype ADHD and predict response to treatment. There is a critical need for multisite prospective carefully designed experimentally controlled or observational studies to identify biomarkers that index inter-individual variability and/or predict treatment response.

## Introduction

Since the earliest times in medicine, doctors and patients have faced key questions, such as which signs and symptoms contribute to establishing a diagnosis, which treatment is likely to be the most effective and least harmful, which course of illness to expect, and if there will be full recovery? Throughout history, answers have been sought and provided through a mixture of natural, spiritual and moral perspectives. Only in the last decades have these questions been addressed by applying systematic measurement of mostly physical, neurocognitive, and behavioral symptoms. The subsequent development of laboratory and imaging techniques to analyze and visualize the interior of the body and the brain have further revolutionized clinical medicine. Today, we are at the transition to an additional level of medical insight known as precision, stratification, or personalized medicine (PM). This perspective paper aims to describe the *status quo* and path toward PM for Attention-Deficit Hyperactivity Disorder (ADHD).

## Precision Medicine

Medicine in the consultation room or at the bedside is directed at an individual patient and thereby *per se* individualized. In a more narrow sense, PM aims to offer evidence-based tailored assessment and treatment to each patient and thereby is an advancing field of healthcare that is informed by each person's unique clinical, genetic/genomic, biomarker, and environmental information (Ginsburg and Willard, 2009). These unique personal characteristics are the basis for treatment, prediction, and prevention. The ambitions of PM therefore go beyond the provision of optimal clinical care and extend to population medicine, screening of high-risk individuals, prevention and personal lifestyle recommendations. In this way, PM forms an integrated, coordinated, evidence-based approach across the continuum from health to disease (Ginsburg and Willard, 2009; Jameson and Longo, 2015). Some prefer the term precision or stratification medicine instead of PM because ultimately recommendations and decisions in clinical practice will be targeted to well defined *groups* of patients with very similar clinical, genetic, or biomarker profiles rather than be directed at the *N* = *1* level.

The goals of PM can be achieved in several ways. The use of diagnostic markers may lead to improved diagnostic accuracy and reduce misclassification, thereby reducing medical shopping, delivering of unnecessary or harmful treatments, and optimizing the use of limited resources. Treatments targeted at the individual level are intended to produce higher response rates, fewer side effects and/or faster responses to treatment and speed up well-being and reduce functional impairment. Successful examples of PM are currently most commonly found in oncology and internal medicine (Hamburg and Collins, 2010; Jameson and Longo, 2015).

However, PM for psychiatric disorders faces several challenges. The most important of these is heterogeneity which is manifest at several levels in psychiatric disorders: phenotypically, with comorbidity being the rule rather than the exception, and aetiologically, with a complex genetic architecture based on different types of genetic variants and gene-environment interplay (Geschwind and Flint, 2015; Boyle et al., 2017), and diverse brain alterations (Faraone et al., 2015). There is no stable, agreed upon, and biologically valid concept of any psychiatric disorder (Kapur et al., 2012), which makes the current taxonomy an unclear basis for informed biological research. Attempts to define more biologically homogeneous subgroups (“biotypes”) (Drysdale et al., 2017) or pathophysiological dimensions of psychiatric disorders are under way (Cuthbert, 2014), but still have to deliver (Wium-Andersen et al., 2017).

## Biomarkers

The success of PM depends on identifying biomarkers, especially stratification biomarkers. A biomarker measures the presence or severity of a condition, its biological subtypes (also called biotypes), course or treatment response; it can be a physical, chemical, biologic, genetic, cognitive, psychological or environmental measure (see Table 1) (Drucker and Krapfenbauer, 2013). Unfortunately, many attempts to identify, validate and implement biomarkers have failed, for example because of lack of (adherence to) rigorous standardized operating procedures, small sample sizes and failures of replication (Drucker and Krapfenbauer, 2013). To be implementable in the clinic, a potential biomarker should be validated across independent data sets, and be reproducible, reliable, specific, sensitive, clinically relevant and feasible (Drucker and Krapfenbauer, 2013; Fusar-Poli et al., 2018).

**Table 1:** Different types of biomarkers.

Diagnostic markers	Predict the presence of a disorder and hence aid its diagnosis
Predictive markers	Assess the most likely response to a particular treatment type
Prognostic markers	Index the course of the disorder over time, with or without treatment
Mechanistic markers	Reflect the underlying pathophysiologic and/or psychological process
Substitute outcome markers	Can be used as surrogate endpoints for a relevant clinical outcome that will be observed later in time
Stratification markers	Subtype heterogeneous disorders into more homogeneous groups

Developing and validating biomarkers requires high-quality study designs. Biomarker discovery may start with retrospective analysis of randomized controlled trial (RCT) datasets for moderator effects. However, most such retrospective analyses are underpowered and/or lack a hypothesis; which is why findings need to be confirmed in prospective designs. The field of oncology has generated advanced designs for testing biomarkers (Freidlin and Korn, 2014). Examples are enrichment designs, in which individuals with a positive biomarker are oversampled and/or biomarker-positive and -negative individuals are balanced; next only biomarker-positive individuals are randomized to a new vs. a standard treatment, or biomarker-positive and biomarker-negative individuals are randomized in a stratified way to new vs. standard treatment. In the latter case, the investigators can be blinded as to the biomarker status of the participants (Mandrekar and Sargent, 2009).

## ADHD

ADHD is a common neurodevelopmental disorder of childhood and has a worldwide prevalence of around 5% (Polanczyk et al., 2007). Persistence into adulthood – either as a full clinical diagnosis or as a subthreshold condition with residual functional impairment is frequent, and adult ADHD has a worldwide prevalence of 2.8% (Fayyad et al., 2017). The categorical diagnosis of ADHD as defined by the DSM-5 (APA, 2013) and ICD-10 (WHO) (WHO, 1992) is based on the presence of pervasive, persistent symptoms of inattention and/or hyperactivity/impulsivity that typically arise early in life and result in significant functional impairment. Current approaches to subtyping in ADHD are limited. The current definition distinguishes three subtypes or presentations, inattentive, hyperactive/impulsive and combined. However, these subtypes appear to be far from stable, and transitions between them are frequent and common (Lahey et al., 2005). A more recent, but equally contentious subgrouping approach for ADHD focuses on age of onset. Contrary to existing definitions which describe ADHD as presenting early in life, there may be a group of individuals in which onset is delayed until adolescence or young adulthood (Asherson et al., 2016; Cooper et al., 2018; Sibley et al., 2018; Asherson and Agnew-Blais, 2019; Breda et al., 2021; Ilbegi et al., 2021; Riglin et al., 2022). However, studies are not consistent as to whether and to what extent late-onset ADHD cases are due to misdiagnosis or underdiagnosis of childhood ADHD symptoms, strong supportive environments in childhood, can be better explained by other (comorbid) disorders, are due to change of rater over time, or are indeed true late-onset cases (Taylor et al., 2021). It is also unclear whether early and late onset ADHD reflect a different balance of genetic and environmental risk factors and share the same underlying neural mechanisms (Asherson and Agnew-Blais, 2019). Pending further study, this could suggest age of onset as a further method for subgrouping patients with ADHD.

There are several critical clinical decision points that may present potential targets for a PM approach to ADHD. The most obvious is predicting treatment response. At present, medication is recognized as the most effective treatment for ADHD (NICE, 2018). However, many clinicians and families prefer to start treatment with non-pharmacological interventions to avoid the potential adverse effects of medication. Unfortunately, meta-analyses have demonstrated limited efficacy for most non-pharmacological treatments by reporting small to modest effect sizes for reducing core ADHD symptoms (Sonuga-Barke et al., 2013; Daley et al., 2014; Cortese et al., 2015, 2016; Oliva et al., 2021; Yang et al., 2021; Groenman et al., 2022). Stronger effects were found on cognitive measures, with modest to large effect sizes for the impact of physical exercise on inhibition and cognitive flexibility (Lambez et al., 2020; Liang et al., 2021). The Achilles heel of most non-pharmacological intervention studies is the lack of blinded raters, lack of an active/sham control group and, even when outcome measures are based on objective laboratory tests, not blinding researchers during data collection and data analysis. Few if any studies have investigated whether there are subgroups of those with ADHD who respond robustly to these treatments.

The main medication treatments for ADHD [methylphenidate (MPH), amphetamines, lisdexamphetamine, atomoxetine (ATX), guanfacine] are associated with moderate to large effect sizes across multiple trials in children older than 6; however, not every individual with ADHD responds well to every medication (Hodgkins et al., 2012), 10–20% of patients do not respond to medication at all, and there is considerable inter-individual variability in tolerability and important adverse effects such as growth problems and hypertension (Faraone et al., 2008). There is currently no clear evidence to guide decision making about who will respond best to and/or tolerate which treatment approach. Further, it is currently not possible to predict course or outcome at the level of the individual patient. It would, for example, be of considerable clinical interest to predict which hyperactive toddlers go on to have full-blown ADHD at school entry, which of those school-age children with ADHD will continue to have problems in adolescence and adulthood, and who will develop which comorbidities and problematic functional outcomes (Caye et al., 2016). Further, while some individuals with subthreshold ADHD symptoms will develop a full clinical syndrome and some may require early treatment, we have no predictors of which subthreshold cases require intensive monitoring and/or treatment (Biederman et al., 2018).

### Systematic Search

A systematic review was undertaken of randomized controlled trials (RCTs) or non randomized studies exploring cognitive, physiological (EEG/ERP), MRI, genetic and clinical measures that may serve as biomarkers to predict diagnosis, stratify ADHD into more homogenous subgroups and/or predict response to treatment and/or predict course (see Supplementary Materials for details of the protocol and search strategy). The search led to the identification of 71 papers, of which 65 were retained after checks for reporting sufficient statistical information. Manual search through PubMed and review papers led to identify another 7 relevant papers. The vast majority of papers reported on EEG (11), cognitive/actigraphic (26) and MRI-based (18) diagnostic markers. Fewer papers described markers to predict response to treatment (11, of which 5 EEG/ERP studies, 3 cognition, 1 NIRS, 1 SPECT and 1 MRI) and biologically subtype ADHD (6) (see Table 2 for a summary of the diagnostic marker studies, and Table 3 for a summary of the other marker studies). The studies are also discussed in the text sections below.

**Table 2:** Diagnostic markers.

**First author (year)**	**Type of biomarker**	**Biomarker**	**Study design**	**ADHD: *N***	**ADHD: Age (y)**	**ADHD: sex ratio (M:F)**	**Controls: *N***	**Controls: Age (y)**	**Controls: sex ratio (M:F)**	**Key findings**
**Neurophysiologic diagnostic markers**										
Chabot and Serfontein (1996)	Diagnostic	QEEG	Case-control study	407	6–17	Not specified	310	6–17	Not specified	Se = 93.7% Sp = 88.0%
Monastra et al. (1999)	Diagnostic	Single–channel QEEG measures: TBR	Case–control study	397	6–30	275:207 (whole sample)	85	6–30s	275:207 (whole sample)	Se = 86% Sp = 98%
Monastra et al. (2001)	Diagnostic	Single-channel QEEG measures: TBR	Case–control study	96	6–20	98:31 (whole sample)	33	6–20	98:31 (whole sample)	Se = 90% Sp = 94%
Li et al. (2005)	Diagnostic	EEG TBR	Case-control study	113	6–14 (10.0 ± SD: 3.0)	88:25	none	none	none	Se = 83.6% Sp = 82.6%
Magee et al. (2005)	Diagnostic	EEG; Total power and absolute and relative power in delta, theta, alpha and beta bands	Case-control study	253	7–13	253:0	67	7-13	67:0	Se = 89.0% Sp = 79.6 % Accuracy 87.0% Cluster analysis found 3 EEG subtypes (maturational lag; hypoarousal; excess beta)
Quintana et al. (2007)	Diagnostic	EEG power bands, TBR, and ADHD-IV rating scale scores (tested against standard psychiatric evaluation)	Case-control study	16	6–15 (*M* 9.8)	14:2	10	6–21 (*M* 11.6)	9:1	EEG: Se = 94% Sp = 100% Accuracy 96% Rating scales: Se = 81% Sp = 22%
Snyder et al. (2008)	Diagnostic	EEG TBR	Clinical cohort study	97	6–18 (10.5 ± 3.4)	101:58	62	n.a.	n.a.	Se = 87% Sp = 94% Accuracy 89%
Liechti et al. (2013)	Diagnostic	EEG	Case-control study	54	8–16 Children *M=*11.1 32–55 Adults *M*= 42.7	31:23	51	*M* 11.2 Children *M* 44.0 Adults		Se = 65% Sp = 60%
Ogrim et al. (2012)	Diagnostic	EEG power bands, TBR	Case-control study	62	7–16	42:20	39	n.a. (age- and sex-matched sample)	24:15	AUC - TBR: 0.63 AUC - Theta: 0.58
Snyder et al. (2015)	Diagnostic	EEG TBR	Prospective clinical cohort study	275	6–18.0 (10.1 ± 2.9) whole sample	64% male, 36 % female whole sample	n.a.	n.a.	n.a.	Se = 0.89 (95% CI: 83–93) Sp: 0.36 (95% CI: 29–44) Integration of the TBR with a clinician's ADHD evaluation raised diagnostic accuracy from 61% to 88%.
Juselius Baghdassarian et al. (2018)	Diagnostic	Auditory brainstem response (ABR)	Case-control study	24	18–50	15:13	63	18–50	30:33	Se = 87.5% Sp = 91.4% AUC: 0.95
**Cognitive diagnostic markers**										
Matier-sharma et al. (1995)	Diagnostic	CPT test + actigraphy	Case-control study	40	6.5–13 (*M* 9.6)	82:29	18	6.5–13 (*M* 9.0)	18:0	Inattention: Se = 70% Sp = 83% Impulsivity: Se = 23% Sp = 88% Activity levels: Se = 25% Sp = 94%
Katz et al. (1998)	Diagnostic	Test of attention span and memory	Case-control study	89	*M* 29.06	21.9 female	20	*M* 35.16		Accuracy: 82.1% (ADHD vs depression)
Grodzinsky and Barkley, 1999	Diagnostic	Neuropsychological tests of frontal lobe functions	Case-control study	66	6–11 years	66:0	64	6–11	64:0	Se = 43% Sp = 93%
Quinn (2003)	Diagnostic (detection of feigning ADHD)	Auditory and visual CPT + ADHD Behavior Checklist scores	Case-control study	16	n.a. (adults)	5:11	42	n.a. (adults)	17:25	CPT differentiates ADHD patients from malingerers: Se = 94% Sp = 91%
Solanto et al. (2004)	Diagnostic	CPT scores	Clinical cohort study	70	34.3 ± 8.9	42:28	33 non-ADHD other psychiatric diagnoses	44.4 ± 10.4	16:17	Se = 47% Sp = 86%
Studerus et al. (2018)	Diagnostic	Neuropsychological tests: Sustained attention and impulsivity in CPT, California Verbal Learning Test), Tower of Hanoi	Case-control study	122	31.6 ± 9.8	77:45	109	25.0 ± 5.3	62:47	Se = 0.73 Sp = 0.77 AUC 0.82 (Differentiating ADHD from high-risk for psychosis)
Gupta et al. (2011)	Diagnostic	Cognitive-motivational tests	Case-control study	120	6–9	Not given	120	6–9	Not given	Accuracy: 97.8%
Jasinski et al. (2011)	Diagnostic	Test of Memory Malingering, Letter Memory Test, Digit Memory Test, Nonverbal Medical Symptom Validity Test, the b Test	Case-control study	38	16:16	19.4/19.78 et al. (2 ADHD groups)	50	18.71/19.41 et al. (2 control groups)		Se = 48% Sp = 100%
López Villalobos et al. (2011)	Diagnostic	Cognitive styles, based on the MFFT-20, CEFT and Stroop tests	Case-control study	100	7–11	158:42 T	100	7–11		Se = 85% Sp = 85%
**Nielsen and Wiig (** **2011** **)**	Diagnostic	AQT processing speed (single- vs. dual-dimension naming speed) and processing efficiency	Case-control study	30	18–43 (28.3 ± 6.6)	14:16	30	n.a. (age- and sex-matched sample)	n.a. (age- and sex-matched sample)	Se = 93% Sp = 100%
Edebol et al. (2012)	Diagnostic	Continuous Performance Test, Motion Tracking System	Case-control study	53	18–64 years	24:29	179	18–53	99:80	Se = 87% Sp = 85%
Ogrim et al. (2012)	Diagnostic	CPT and Go-NoGo task	Case-control study	62	7–16	42:20	39	n.a. (age- and sex-matched sample)	24:15	Omission errors: Se = 85% Sp = 85%
Wiig and Nielsen (2012)	Diagnostic	AQT processing speed (single- vs. dual-dimension naming speed), processing efficiency	Retrospective case-control study	64	17–54 (*M* 31.1)	36:28	30	18–43 (*M* 28.3)	16:14	Se = 80%−89% Sp = 100%
Zelnik et al. (2012)	Diagnostic	ADHD scores from the Variables of Attention Test	Case-control study	179	6.0–17.5 (10.0 ± 2.7)	2.4:1	none	none	none	Se = 91.1 % Sp = 21.6 %
Edebol et al. (2013)	Diagnostic	Qbtest	Case-control study	Clinical ADHD = 55 ADHD Normative = 84	Priority:	25:30 47:37	Non-ADHD normative = 202	18–53	114:88	Se = 86% Sp = 83%
Esposito et al. (2016)	Diagnostic	Eye-movement assessing eye vergence in orienting attention	Case-control study	Trained model testing = 19 Validation set = 22	7–14	Not given	Trained model testing = 19 Validation set = 210	7–14	Not given	AUC: 0.95 Accuracy: 90.8%
Fuermaier et al. (2016)	Diagnostic	Embedded Figures Test	Case-control study	51	22–45 (34 ± 11.3)	30:21	Healthy comparison group = 52 Psychology student control group = 58	23–46 18–25	24:38 22:36	Se = 88% Sp = 90% AUC (feigned ADHD vs actual ADHD): 94.8%
Gilbert et al. (2016)	Diagnostic	Actigraphy + CPT	Case-control study	70	7.6 ± 11.2	64:6	70	7.6 ± 10.7	64:6	Se = 81.4% Sp = 91.4%
Groom et al. (2016)	Diagnostic	Qbtest	Case-control study	ADHD = 37	18–60 years	24:13	ASD = 25	19–47	19:6	Se = 84% Sp = 76% AUC: 0.87 (differentiate ADHD from ASD)
Fuermaier et al. (2017)	Diagnostic	Visuospatial WM test	Case-control study	Visuospatial WM = 48 Cross validation sample = 27	45–23 (34.2 ± 11.3) 21–45 (33.5 ± 11.7)	27:21 17:10	Visuospatial WM (HCG) = 48 Stimulation design (CG) = 48	22–46	22:26 17:31	Se = 60.3% Sp = 95.8% AUC: 0.90
Hollis et al. (2018)	Diagnostic	Qbtest	Diagnostic RCT	267 (132 = intervention arm, 135 = control arm)	6–17	Intervention arm = 95:28 Control arm = 102:25	N/a	N/a	N/a	Qbtest: faster diagnostic decision at 6-months (76% vs. 50%), shortening appointment time by 15%, increased clinicians' confidence in diagnostic decisions (odds ratio 1.77, 95% CI 1.09–2.89)
Hult et al. (2018)	Diagnostic	Qbtest	Case-control study	124	*M* 10.3	97/27	58 – other Clinical Diagnoses	*M* 10.8		Se = 47–67% Sp = 72–84% AUC: QbInattention and QbActivity = 0.76 and.074
Cohen et al. (2019)	Diagnostic	Graphology	Case-control study	22	13–18	15:7	27	13–18	6:21	Girls: Se = 71.4% Sp = 80.0% Boys: Se = 76.2% Sp = 75.0%
Unal et al. (2019)	Diagnostic	Response time and accuracy in 3 visual attention tests (Stroop test, Stroop Effect test with visual aid, Perceptual Selectivity test)	Case-control study	14	18–65 (47.3 ± 9.0)	9:5	30	41.6 ± 11.4	13:17	Stroop test (accuracy and response time) best separated ADHD from controls accuracy: AUC 0.814, 95% CI: 0.679 – 0.949 response time: AUC, 0.810, 95% CI: 0.664–0.955
Berger et al. (2021)	Diagnostic of feigned ADHD	MOXO-d-CPT	Retro-spective	47	18–65	17:30	47	18–65	14:33	Attention index: Se = 61.0% Sp = 91.5% AUC: 0.93
Johansson et al. (2021)	Diagnostic	Qbtest	Case-control study	89	63:26	*M* – 15	251	*M*-15		Se = 67.1% Sp =58.4% AUC: 0.48–0.64
**Brain imaging based diagnostic markers**										
Soliva et al. (2010)	Diagnostic	MRI Caudate body volume (rCBV/tbCV and rCBV/bCBV ratio)	Retrospective case-control-MRI study	39	6–16 (10.9 ± 2.8)	35:4	39	6–17 (11.5 ± 2.9)	Received Date: 19-May-2022	Se = 42.1 % Sp = 94.7 % AUC 0.84 (95% CI = 0.69–0.94)
Bohland et al. (2012)	Diagnostic	Anatomical MRI attributes and local and global resting state fMRI measures	Case-control study	272	7–21	219:53	482	7–21	255:227	Network features: AUC: 0.78 (test set)
Cheng et al. (2012)	Diagnostic	Multiple neuroimaging markers	Case-control study	98	12.1 ± 2.1	91:10*^1^	141	11.4 ± 1.9	84:59	Se = 63.3% Sp = 85.1%
Colby et al. (2012)	Diagnostic	Multiple structural and functional MRI features	Case-control study	273	7–21	215:58	341	7–21	160:181	Se = 33% Sp = 80%
Dai et al. (2012)	Diagnostic	Resting-state functional connectivity	Case-control study	22	11.3 ± 3.0	173:48	402	12.47 ± 3.39	208:194	Se = 31.4% Sp = 71.8% AUC: 0.70
Eloyan et al. (2012)	Diagnostic	Resting state functional connectivity and structural brain imaging	Case-control study	285	Review Article	38:62	491 Internal test set = 262 Internal training set = 363	7–26	38:62	Se = 21% Sp = 94%
Sidhu et al. (2012)	Diagnostic	Phenotypic and resting-state fMRI data (SVM classifier with machine learning)	Machine learning based experimental design	239	n.a.	n.a.	429	n.a.	n.a.	Phenotypic + fMRI data: Accuracy: 0.76
Lim et al. (2013)	Diagnostic	MRI grey matter volumetric data	Case-control study	29		29:0	29	10.7–17.9 *M*= 14.4		Se = 75.9%, Sp = 82.8%. AUC: 0.83
Dey et al. (2014)	Diagnostic	Resting state fMRI	Case-control study	487	8–26	307:180	307	8–26	Not specified	Training dataset: Se = 30.7% Sp = 84.7%
Hart et al. (2014)	Diagnostic	Task based fMRI tasks (inhibition)	Case-control study	30	10–17		30	10–17	30:0	Se = 90% Sp = 63% Accuracy: 77% AUC: 0.81
dos Santos Siqueira et al. (2014)	Diagnostic	Resting state fMRI signals: graph-derived centrality measures, classified by a linear SVM algorithm	Machine learning based experimental design	269	11.6 ± 2.9	215:54	340	11.6 ± 2.9	180:160	Se = 31%-50% for 3 different measures) Sp = 62%-74% for 3 different measures
Ishii-Takahashi et al. (2014)	Diagnostic	Oxygenated Hb concentration changes in the PFC during a stop signal task and a verbal fluency task	Case-control study	Word	*M* 30.6	11/8	21	*M* 28.8		Se = 84.2% Sp =76.2% accuracy = 78.8% Leave one-out classification algorithm
Monden et al. (2015)	Diagnostic	Spatial distribution and amplitude of hemodynamic response in multichannel fNIRS	Case-control study	30	6–15 (9.1 ± 2.6)	20:10	30	6–14 (9.7 ± 2.3)	25:5	Se = 90.0 % Sp = 70.0 % AUC: 0.85
Kessler et al. (2016)	Diagnostic	Resting-state fMRI Intrinsic Connectivity Network analysis	Case-control study		*M* 14.8	16:8	494	*M* 15.7		Components A-C significantly predicted ADHD diagnosis (ORs 1.70 to 2.07)
Gehricke et al. (2017)	Diagnostic	Morphometric MRI	Case-control study	32	19–31 (25.3 ± 5.4)	26:6	40	20–28	33:7	AUC: 0.92
Sen et al. (2018)	Diagnostic	MRI structural features and functional connectivity from resting state fMRI scans (used to create a machine learning model)	Machine learning based experimental design; cross-validation and holdout set evaluation	training set: 279, hold-out set: 77	n.a.	n.a.	training set: 279 hold-out set: 94	n.a.		Multimodal features (MRI + fMRI): Hold-out: Se = 45.5% Sp = 85.1% Replication in ABIDE: Se = 60.0% Sp = 68.3%
Chen et al. (2019)	Diagnostic	Multiscale functional brain connectomes (anatomical and functional MRI) + personal data	Retrospective case-control	246	11.4 ± 2.46	193:53	346	11.8 ± 2.64	192:154	AUC: 0.82 (95% CI: 0.8–0.83)
Kautzky et al. (2020)	Diagnostic	PET imaging of SERT and genotypes of HTR1A, HTR1B, HTR2A and TPH2 genes	Case-control study	16	*M 31.9*	7:9	22	*fnbeh-16-900981*		Se = 0.82 Sp = 0.80 Accuracy: 0.80

**Table 3:** Treatment response and subtyping markers.

**First Author (year)**	**Type of biomarker**	**Biomarker**	**Study design**	**ADHD: *N***	**ADHD: Age (y)**	**ADHD: sex ratio (M:F)**	**Controls: *N***	**Controls: Age (y)**	**Controls: sex ratio (M:F)**	**Key findings**
**Treatment response markers**										
Young et al. (1995)	Treatment response to stimulants	ERP response (P3b amplitude changes) to a single dose administration of MPH (via Auditory Oddball Task)		26	13.3 ± 2.5	15:11	–	–	–	The post drug changes in P3b amplitude separated MPH responders and non-responders: Se = 86%, Sp = 75% Accuracy: 81%
Chabot et al. (1999)	Treatment response to stimulants	QEEG	Retrospective	130	6–17	97.5:32.5	310	6–17	Not specified	Se = 83.1% Sp = 88.2%
Hermens et al. (2005)	Treatment response to stimulants	Oddball test and WM test	Pre-post stimulant treatment	50	9–18 years (13.6 ± 1.9)	40:10	–	–	–	Oddball test: accuracy 85.0% of responders and 95.0% of non-responders. WM test: accuracy 80.0% of responders and 90.0% of non-responders
Cho et al. (2007)	Treatment Response to stimulants	Regional cerebral blood flow (rCBF) with SPECT	Pre-post treatment	34	8.4 ± 2.5	30:4	–	–	–	Se = 88.2%
Cho et al. (2007)	Treatment response to stimulants	Variability of Response Time (RT)	Pre-post treatment	144	6–18 *M* 9.3	–	–	–	–	Variability of RT predicted 94.9% of responders, 17.2% of non-responders (overall 69.3%)
Johnston et al. (2015)	Treatment response to stimulants	Clinical and neuropsychological measures	Pre-post treatment	43	43:0	*M* 11.2 responders *M* 11.3 non-responders	–	–		Se = 54% Sp = 87% Accuracy: 77%
Kim et al. (2015)	Treatment response to stimulants	ADHD Rating Scale-IV and Disruptive Behavior Disorder rating scale, CPT, Stroop test, resting-state fMRI scans. Polymorphisms of DAT, DRD4, ADRA2A and NET genes	Pre-post treatment	78	*M* 9.4/9.8 et al. (2 ADHD groups)	62:16	–	–		Accuracy: 84.6% AUC: 0.84
Ishii-Takahashi et al. (2014)	Treatment response to stimulants	Near-infrared spectroscopy (NIRS).	Pre-post treatment	30	M 8.6	26/4	20			Se = 81.3% Sp = 80% Accuracy: 81%
Arns et al. (2018)	Treatment response to stimulants	Resting-state EEG measures: Alpha peak frequency (APF) and Theta/Beta ratio (TBR)	Pre-post treatment	336	M 11.9 ± 3.3	245:91	158	*M 12.2* ±*3.2*	112:46	Male (but not female) non-responders had low frontal APF. AUC: 0.71 (resp) AUC: 0.29 (non-resp). Response was not associated with TBR
Griffiths et al. (2017)	Treatment response to atomoxetine	ERPs to auditory oddball task	Double-blind placebo-controlled cross-over trial	52	6–17 years M 11.9 ± 2.5	43:9	52	Age and sex-matched	Age and sex-matched	Responders had significantly lower auditory oddball N2 amplitudes particularly in the right frontocentral region. Se = 58.8% Sp = 80.8%
Norman et al. (2021)	Treatment response to stimulants	Resting-state (rs) MRI connectivity	Pre-post treatment	110 (196 observations)	6–17 years M 10.8 ± 2.2	110:28	142 (330 observations)	6–17 years M 10.5 ± 2.8	142:61	Worse response to treatment was associated with increased cingulo-opercular connectivity with increasing age
**Subtyping markers**										
Magee et al. (2005)	Subtyping	EEG; Total power and absolute and relative power in delta, theta, alpha and beta bands	Case-control study	253	7–13	253:0	67	7–13	67:0	Cluster analysis found 3 EEG subtypes (maturational lag; hypoarousal; excess beta)
Fair et al. (2012)	Subtyping	20 Neuro-psychological tests that covered inhibition, working memory, arousal/activation, response variability, temporal information processing, memory span, and processing speed	Case-control study	244	6–17	68% male	213	7–17	42.3% male	Community detection analyses found 4–6 cognitive subgroups, both among the ADHD and the control sample
Coghill et al. (2014)	Subtyping	Neuropsychological measures of matching to sample, spatial working memory, decision making, inhibition, choice delay,	Case-control study	83	6–12 8.9 ± 1.7	Boys only	66	6–13 9.0 ± 1.7	Boys only	Confirmatory Factor Analysis found six latent cognitive factors (working memory, inhibition, delay aversion, decision making, timing, variability)
van Hulst et al. (2015)	Subtyping	Neuropsychological measures (12 measures) of cognitive control/timing, reward sensitivity,	Case-control study	96	4	76:20	121	6–25 13.6 ± 4.3	89:32	Latent class analysis found 3 subgroups (covering 87% of ADHD sample: quick and accurate; poor cognitive control; slow and variable timing)
Leikauf et al. (2017)	Subtyping	Neuropsychological measures of attention, inhibition, switching, spatial memory, verbal memory. digit span, reaction time, interference	Case-control study and placebo-controlled RCT with atomoxetine	116	6–17	90:26	56	n.r.	n.r.	Cluster analysis found 2 ADHD subtypes (impulsive cognition; inattentive cognition) that differed in terms of EEG characteristics and response to atomoxetine
Mostert et al. (2018)	Subtyping	Neuropsychological measures of working memory, attention, inhibition, set-shifting, verbal fluency, delay discounting, time estimation	Case-control study	133	M 35.6 ± 10.4	42% male	132	19–63 M 36.3 ± 11.6	40% male	Community detection analyses identified 3 subtypes (altered attention and inhibition; increased delay discounting; altered working memory and fluency)

The next sections incorporate the results of the search and provide short state-of-the-art summaries of what is known regarding markers for diagnosis and treatment selection in ADHD.

### Clinical Aspects

Inattention and hyperactivity/impulsivity symptoms follow specific developmental courses and have different relationships to co-occurring disorders. The data are mixed as to whether the nature of presenting symptoms predicts differential response to treatment with medication (Solanto et al., 2009). Severity of ADHD symptoms and associated oppositional defiant disorder or conduct disorder may be associated with worse response to treatment (Chazan et al., 2011). Comorbid anxiety and depressive disorders are not associated with reduced effects of stimulants on ADHD symptoms (Gadow et al., 2002). Children with associated tic disorders or autism spectrum disorder (ASD) also have a favorable response to stimulants (Bloch et al., 2009; Reichow et al., 2013). However, the reduction of ADHD symptoms in children with tic disorders and ASD is generally smaller than reported for ADHD without tics or ASD (Cortese et al., 2018), and emergence of tics and adverse events are more common in these populations (Bloch et al., 2009; Reichow et al., 2013). Comorbid bipolar disorder (BD) and substance use disorder (SUD) are associated with increased severity of ADHD and generally of poorer prognosis (Goldstein et al., 2017; Luo and Levin, 2017). The presence of these disorders does not preclude the use of stimulants, but when offered, stimulant treatment should be preceded by mood stabilizers for those with BD and assessment of abuse liability and drug interactions for those with SUD. In adults with ADHD, comorbidity with personality disorders is associated with a lower response rate to stimulants and a more complex clinical course (Robison et al., 2010). Subtyping of ADHD by interindividual differences in temperament and in particular by emotional regulation/dysregulation has been proposed as a promising way to parse the disorder's clinical and cognitive heterogeneity and develop models to predict treatment response and course (Karalunas et al., 2014, 2019; Nigg et al., 2020).

### Genetics and Pharmacogenetics

ADHD is a highly heritable disorder with a complex genetic architecture (Faraone and Larsson, 2019; Brikell et al., 2021). Most cases seem to be due to multiple genetic variants (of mostly limited effect size), in interaction with environmental risk factors. Given this polygenic nature of ADHD, use of genetic testing is still limited in diagnostic assessments of ADHD. However, recent evidence suggesting a role for more penetrant rare genetic mutations in a subgroup of patients (Demontis et al., 2016; Kim et al., 2017; Ganna et al., 2018) may lead to an increased use of genetic diagnostic testing. If their accuracy can be dramatically improved, polygenic risk scores (PRS) may become important for polygenic ADHD cases, since PRS have been shown to predict symptom severity and comorbidity in ADHD (Hamshere et al., 2013; Stergiakouli et al., 2015; Ronald et al., 2021) and are informative regarding symptom persistence (Riglin et al., 2016; Agnew-Blais et al., 2021). Epigenetic profiling of a set of ADHD risk genes was claimed to predict ADHD among Chinese children with 93% accuracy but this has not been replicated (Xu et al., 2015).

Pharmacogenetics is the study of genetic variability in medication response. Numerous studies of stimulant response have been conducted, primarily with genes related to catecholamine functions (Froehlich et al., 2010). Effect sizes have been small, and few consistent findings have been reported (Hart et al., 2013; Myer et al., 2017). Given the poor adherence to ADHD medications, perhaps a more appropriate target for pharmacogenetic studies is tolerability (Joensen et al., 2017). Despite a paucity of studies and large between-study heterogeneity, associations have been found between adverse events and several genetic variants (see Table 4). Indeed, maintenance of effects and tolerability are key-drivers of long-term outcome and may be more salient targets for genetic studies than acute symptom reduction. Since the majority of common adverse events are dose-related, pharmacogenetic studies associated with inter-individual variability in drug metabolism hold considerable promise, such as CYP2D6 for amphetamines and ATX (Bach et al., 1999; Yu et al., 2016), and Carboxylesterase-1 gene (CES1) which is the sole enzyme involved in MPH metabolism (Stage et al., 2017). A regularly updated database of pharmacogenetic findings can be accessed via the Clinical Pharmacogenetics Implementation Consortium: https://cpicpgx.org/.

**Table 4:** Associations between adverse events to ADHD medication and genetic variants (Joensen et al., 2017).

**Adverse events**	**Genetic variants**
Appetite reduction	CES1*G
Buccal-lingual movements	T1065G
Diastolic blood pressure	ADRA2A Mspl C/C-GC
Emotionality	DAT1*9/9
Irritability	SNAP25 T1065G
Picking	DRD4*7/DRD4*4
Social withdrawal	DRD4*7/DRD4*4
Somatic complaints	DAT1*10/10
Tics	5-HTTLRP*S/L*L/L; SNAP25 T1065G
Sadness	CES1*rsl12443580
Vegetative symptoms	5-HTTLPR

### Metabolomics and Neurochemistry

A meta-analysis reviewed 71 studies of metabolites in monoaminergic transmission pathways, 18 studies of heavy metals, 16 studies of substance or chemical exposures, 29 studies of trace elements, 24 studies of essential fatty acids, 22 studies of hypothalamic pituitary axis pathway metabolites, 15 studies of growth and thyroid function, 7 studies of oxidative stress, and 3 studies of cytokine imbalance (Scassellati et al., 2012). Because the lack of availability of brain tissue in ADHD, these were all studies of peripheral samples: plasma, serum, urine, saliva, blood, platelets, red blood cells, cerebrospinal fluid, and hair. Of all biomarkers meta-analyzed, eight were statistically significantly associated with ADHD after correcting for multiple comparisons: norepinephrine (NE) in urine, 3-methoxy-4-hydroxyphenylethylene glycol (MHPG) in urine, monoamine oxidase (MAO) in platelets, normetanephrine (NM) in urine, metanephrine (MET) in urine, ferritin (FE) in serum, zinc (ZN) in serum/plasma/urine, and cortisol in saliva. NE, MHPG, MAO and cortisol were also associated with drug response and ADHD symptom severity. Given the consistency of findings for NE, MHPG and MAO, it is possible that lower MAO activity interferes with the degradation of NE leading to lower levels of MHPG. Urinary “MAO-NE-MHPG” levels could provide a biochemical marker profile for ADHD (Scassellati et al., 2012). Yet, because prior studies of these biomarkers have focused on group effects rather than on diagnostic accuracy and individual prediction, more work is needed to define a valid and accurate biomarker profile from this triad. A study observed serum levels of sphingomyelins to be significantly lower in ADHD patients compared to controls with a separation at the individual level with 79% sensitivity and 78% specificity (Henríquez-Henríquez et al., 2015).

### Neurophysiology

Electroencephalogram (EEG) markers such as theta and beta oscillations at rest reflecting arousal, alertness, orienting and activation (Clarke et al., 2013; McLoughlin et al., 2022) and task-related cognitive event-related potentials (ERPs) reflecting preparation (contingent negative variation, CNV), attention and inhibition (earlier P100, N100, P200, N200 and later P300 components) reveal moderate deviations mainly for later ERPs and heterogeneity in ADHD at the level of functional brain systems (Kaiser et al., 2020). Consistent subgroups based on these EEG markers include clusters showing increased theta and reduced beta activity, increased slower activity resembling what is seen in developmental delay, or increased beta activity; fewer consistent subgroups were reported for ERPs (Barry et al., 2003). These markers partly cluster independently of clinical DSM-based subtypes and comorbid diagnostic categories (Mazaheri et al., 2014).

The search identified 11 studies that used EEG-based measures and in particular the power of the frequency bands and the ratio of the theta to beta power (TBR) to predict a diagnosis of ADHD in case-control designs (see Table 2). TBR was initially hypothesized to be a measure of central arousal; later, it was reconsidered to be more an index of cognitive processing and executive control (Clarke et al., 2019). The EEG diagnostic work however suffers from a lack of replication in independent large samples and standardization of data collection (eye open vs. eyes closed condition) (Clarke et al., 2020). Further, the results of the various studies differ but, in terms of sensitivity and specificity, mostly fall behind criteria for application as clinical test (accuracy > 0.90). In terms of subtyping ADHD, cluster analysis of resting-state EEG data showed three clusters (maturational lag, hypoarousal, and excess beta-hyperarousal) (Magee et al., 2005) but differences in event-related potentials (ERPs) between these clusters on a separate odd-ball task were only minimal (Brown et al., 2005).

Longitudinal work has identified consistent neurophysiological patterns related to differential outcomes. Children with ADHD persisting into adulthood show increased beta and reduced frontal theta EEG at rest (Clarke et al., 2011), and ERP markers for reduced cognitive preparation and error processing (Doehnert et al., 2013; Cheung et al., 2016; Michelini et al., 2016).

Studies predicting treatment response using EEG-based markers have focused on neurofeedback and medication. Neurofeedback studies indicate that increased theta predicts response to theta-modulating neurofeedback (Gevensleben et al., 2009; Janssen et al., 2016), and parietal alpha plus a strong CNV reflecting cognitive preparation together explain nearly 30% of the variance in slow cortical potential neurofeedback outcome. Recent work describes that a high individual peak frequency (iAPF) of the EEG is associated with higher remissions rates both to neurofeedback and medication treatment (Voetterl et al., 2022). Medication studies report that increased theta and absence of frontal slowing is associated with better stimulant response in some (Chabot et al., 1999; Clarke et al., 2002; Arns et al., 2008) but not all (Janssen et al., 2016) studies. Changes of the P3b component of the ERP after administration of a single dose of MPH separated medication responders and non-responders with 81% accuracy (Young et al., 1995). In the largest study to date, theta/beta ratio did not predict MPH response, and frontal alpha slowing predicted non-response only in a male adolescent subgroup (Arns et al., 2018). The response to ATX was predicted by stronger cognitive N2 activity (Griffiths et al., 2017). The EEG and ERP markers predicting pharmacological and non-pharmacological treatment response partly overlap. However, given the limitations of retrospective analyses of treatment response, independent and large controlled prospective multicenter studies with guideline based diagnosis and treatment are needed to clarify the promise of EEG based marker for PM in ADHD (Dopfner et al., 2017).

### Neurocognition

Many studies have used the omission and commission error scores of a Continuous Performance Task (CPT) often in combination with actigraphy or movement assessments to index the inattention, impulsivity and hyperactivity symptom domain to develop cognitive diagnostic markers predicting ADHD (Table 2). Other measures used are tests of working memory, attention, other executive functioning tests, eye vergence to index visual orienting, reaction time characteristics, and quality of handwriting evaluated by a blinded graphologist (Table 2). Cognitive tests have also been explored for differentiating “true ADHD” from feigned ADHD and malingering (Quinn, 2003; Berger et al., 2021). Predictive statistics vary and for a couple of studies comes close to being clinically relevant. Stringent replication in independent samples however is lacking. A diagnostic randomized single-blind study showed that the Qbtest (a combination of CPT and movement analysis by an infrared measurement system) as an add-on to the standard clinical assessment approach did improve clinical decision-making and was associated with faster diagnostic decisions, a shorter clinical consultation time and higher clinician confidence in the diagnosis, compared to standard clinical assessment only (Hollis et al., 2018).

Psychological factors such as compliance, suggestibility, motivation, coping strategies, and cognitive capacities are all important predictors of treatment success and should be considered when taking a whole person approach to PM (Cutica et al., 2014). Several approaches to stratification of ADHD with the aim of producing more homogenous neurocognitive subgroups have been described ADHD (Fair et al., 2012; Coghill et al., 2014; van Hulst et al., 2015; Leikauf et al., 2017; Mostert et al., 2018). The most ambitious study applied unsupervised machine learning on a battery of cognitive tests in 116 children with ADHD and identified two biotypes that were validated by EEG and ECG measures and differential response to medication. One biotype was labeled impulsive cognition and characterized by high rates of commission errors and shorter reaction time, greater EEG slow wave (theta/delta) power and greater resting heart rate. The second biotype was labeled inattentive cognition and was marked by high rates of omission errors, longer and more variable reaction time and lower EEG fast wave (beta) power. The biotypes did not differentially predict the clinical response to atomoxetine, but only within the inattentive cognitive biotype a strong correlation was observed between change in ADHD symptoms and change in cognitive measures such as verbal memory immediate recall (Leikauf et al., 2017). This approach is informative for PM approaches to predict treatment response for both medication and non-pharmacological (cognitive) interventions. Further research could lead to matching specific cognitive and educational interventions to particular cognitive deficits (van der Donk et al., 2013). Moreover, neurocognitive predictors of longitudinal course and outcome in ADHD may give hints to PM approaches. A study investigated the predictive value for ADHD course and severity of eight cognitive domains; the authors reported better baseline working memory to be correlated with less ADHD severity at follow up 6 years later, whilst reduced reaction time variability was associated with better global functioning (van Lieshout et al., 2017).

Studies on the prediction of treatment response from neurocognitive measures are limited. Performance on an oddball and working memory test (Hermens et al., 2005) and variability of response time (Lee et al., 2009) did separate responders from non-responders to stimulants with about 85 and 70% accuracy respectively. Machine learning analyses on a large set of pre-treatment measures learned that the performance on the Stroop test together with information on candidate genes polymorphisms and clinical characteristics predicted stimulant response with 84.6% accuracy (Kim et al., 2015). Performance on a delayed matching to sample task (Coghill et al., 2007) and on the go/no go task were significantly associated with MPH response (Johnston et al., 2015). For future research, it will be important to integrate neurocognitive data with other biomarkers across the different levels of analysis. Further, it is important to focus on neurocognitive tasks that are conceptualized from a neuroscience perspective, and have shown to be dependent on particular neuroanatomical, and neurophysiological substrates, such as the CANTAB battery of tasks (Fried et al., 2015).

### Brain Imaging

Many studies have attempted to predict ADHD diagnoses from neuroimaging data, with some claiming high accuracy (see Table 2). Most studies performed various sorts of machine learning using features extracted from resting-state MRI, structural MRI or a combination of these, while one used task-based MRI, one the hemodynamic response assessed by fNIRS, and another a combination of PET-scan imaging of the serotonin-transporter and serotonergic genes. Methodological limitations such as mostly small sample sizes and lack of out of sample validation lead to adjust these claims (Wolfers et al., 2015). The largest predictive study derives from the ENIGMA ADHD consortium's structural MRI data and was able to separate ADHD from non-ADHD individuals at the group level by machine learning, but not with sufficient specificity to be considered clinically useful for individual prediction (Zhang-James et al., 2021).

Few studies have examined predictors of treatment response from brain imaging data. Resting-state regional blood flow measured by SPECT pre-treatment (Cho et al., 2007) and by hemodynamic response to single doses of stimulants assessed by NIRS (Ishii-Takahashi et al., 2014) were associated with the response to stimulants. Robust improvement with MPH was associated with greater reduction in dopamine transporter (DAT) density in putamen, measured by Tc-99m] TRODAT-1SPECT (Akay et al., 2018). Other research used imaging together with genotype and clinical/demographic factors to determine the brain basis of improvement with MPH treatment; in younger children and lower treatment levels, carriers of the DRD4 7R allele showed decreased frontal cortex volumes - suggesting that youth with the DRD4 7R genotype might be sensitive to cortical remodeling after low dose stimulant treatment (Schweren et al., 2016). Another study examined the activation profiles of fMRI scans obtained while performing an inhibitory control task off treatment to predict response to treatment with MPH and ATX (Schulz et al., 2017). Improvement with ATX was predicted by relatively higher activation in motor cortex off medication, while superior response to MPH over ATX was predicted by elevated caudate activation (Schulz et al., 2017). A longitudinal study of resting-state connectivity and response to stimulant treatment found that decreasing connectivity within the cingulo-opercular network with increasing age was associated with a better treatment response to stimulants (Norman et al., 2021).

Another application of the PM approach has used structural MRI to characterize ADHD symptom profiles and/or to predict persistence vs. remission over time. In longitudinal designs, persistent ADHD relative to remitting ADHD was associated with a thinner cortex in the medial occipital cortex, insula, parahippocampus, and prefrontal regions (Proal et al., 2011). Adults with persistent ADHD were characterized by greater cortical thinning in the medial and dorsolateral prefrontal cortex; moreover, the rate of cortical thinning was correlated with the number of inattention symptoms (Shaw et al., 2013). Loss of cerebellar volume over time was further associated with worse outcome (Mackie et al., 2007). A study using longitudinal voxel-based morphometry found that low volume in fronto-cerebellar brain regions involved in cognitive control and larger amygdala volume were associated with persistence of ADHD, while low volume in the visual and auditory cortices was associated with remission over time (Adamo, 2018).

Given the variability in phenotypic expression across individuals with ADHD, imaging studies may have their greatest utility for determining biomarkers of subgroups within the larger ADHD population, which may have predictive value in relation to course, differential response and tolerability, and possibly even dose. Three studies have made a start by using resting-state MRI data to biologically subtype ADHD using different processing and data analytic pipelines (Gates et al., 2014; Costa Dias et al., 2015; Lin et al., 2018). Further work in this direction including out of sample validation is needed.

### Environmental Factors

Multiple indicators of psychosocial adversity, including family adversity, low income, and low socioeconomic status are associated with ADHD (Dopfner et al., 2008; Thapar et al., 2013; Russell et al., 2016). Maternal nicotine and alcohol use during pregnancy and exposure to pesticides and lead may influence the development of ADHD symptoms (Linnet et al., 2003; Sciberras et al., 2017). However, many of these environmental effects are not specific to ADHD and/or may disappear when genetic influences are controlled for (Rice et al., 2018). Extreme forms of early deprivation can result in ADHD-type symptoms (Rutter et al., 2007; Kennedy et al., 2016). Studies examining the influence of psychosocial adversity on pharmacological, psychosocial or combined treatment have produced unclear results (Handen et al., 1994; Thomson and Varley, 1998). Single-parent status, low income, and education are associated with a poorer behavioral treatment response in children with ADHD, lower adherence, and higher attrition for treatments (Firestone and Witt, 1982). Poor adherence to pharmacological treatment is predicted by high maternal psychological distress, indifferent parenting, maternal overprotection/control, poor family support, decreased interaction with parents, and increased problems at home (Gau et al., 2006).

Moderator analyses conducted in the Multimodal Treatment Study of ADHD (MTA) showed that, for families from the lowest socioeconomic strata, who required welfare support, only Combined Treatment of medication and psychosocial intervention yielded meaningful benefits with respect to the outcome of teacher-reported social skills (MTA Cooperative Group, 1999). For children receiving either medication or combined treatment, caregiver symptoms of depression predicted *worse* outcome than for those with less-depressed parents (Owens et al., 2003; Hinshaw, 2007). In behavioral interventions for children with ADHD, parenting self-efficacy plays a moderating role in decreasing comorbid behavioral problems, but not in decreasing ADHD symptoms (Hinshaw, 2007). In behavioral interventions for children with ADHD parenting self-efficacy plays a moderating role in decreasing comorbid behavioral problems, but not in decreasing ADHD symptoms (van den Hoofdakker et al., 2010).

### Sex

ADHD is more common in boys than girls, and the male preponderance is higher for hyperactivity/-impulsivity than for inattentiveness (Greven et al., 2018). In adulthood, the sex ratio is more even (Greven et al., 2018). It is unclear, whether course and long-term prognosis differ between boys and girls with ADHD. Although sex is an important aspect in the clinical manifestation of ADHD, research on PM according to sex is relatively immature (Greven et al., 2018). Sex-specific aspects of treatment include a focus on sex-specific comorbidities as well as treatment response. For example, adolescent girls with ADHD have increased risk for early sexual activity, sexually transmitted diseases and teenage pregnancy, compared to girls without ADHD (Heerde et al., 2015; Snyder, 2015). Up to now, it appears that sex-specific factors do not play a strong role in the pharmacological and non-pharmacological treatment of ADHD. The safety and response to medication is overall comparable between males and females with ADHD (Storebo et al., 2015), but see (Sonuga-Barke et al., 2007). In the context of PM it is important to train teachers and professionals in recognizing sex-specific manifestations of ADHD.

### Patient Preferences

It is important to take patients' preferences into account, since patients/families receiving the treatment of their choice tend to show better treatment adherence and clinical outcomes (Swift et al., 2011). Patient preference is particularly relevant in PM for ADHD since novel treatment options, such as nutritional interventions, computerized cognitive training, neurofeedback, and mindfulness training are increasingly considered (Stevenson et al., 2014; Cortese et al., 2015, 2016), and weighing the pros and cons of the various treatment options becomes more complex. Several methods are available that can help clarify treatment preference, such as the discrete choice experiment or the best-worst scaling method (Schatz et al., 2015).

### Health Economics

ADHD has a profound negative impact on costs for health care both at the individual and the societal level (Le et al., 2014). Misdiagnosis or selection of an intervention not suitable or harmful to the individual patient will compromise cost-effective treatment of ADHD, as ineffective treatments and wastage of resources boost health care expenditures (Akhmetov et al., 2015). Further, an initial diagnosis at a much older age that had been missed earlier is associated with substantially higher health care and societal costs (Daley et al., 2015). PM holds the potential to lower health care-related costs, if patients immediately receive those therapies and interventions that are most likely be safe and effective for them (Abadi-Korek et al., 2013). However, to remain profitable for manufacturers, targeting smaller subpopulations identified by specific assessment methods, e.g. genomic testing, with highly specific therapies may also increase treatment expenses (Faulkner et al., 2012). Across Europe and the United States, there is significant variation and fragmentation with respect to regulations regarding licensing and reimbursement of PM technologies, and national health care systems are limited in their ability to adequately evaluate and provide access to personalized diagnostics and treatments brought to the market (Akhmetov et al., 2015). Reliable guidance for the design of studies that combine regulatory and reimbursement needs is lacking for novel PM technologies in many countries, though there is guidance from the Federal Drug Administration for clinical pharmacogenomics studies (FDA, 2013). Such guidance is mandatory for industry to gain a clear picture of the evidence required to receive market authorization and reimbursement within the public insurance systems. Clarifying areas of uncertainty and moving toward standard regulatory and reimbursement practices on a multi-national level will facilitate the broader adoption of PM into clinical practice (Faulkner et al., 2012).

### Ethical Considerations

PM inevitably also faces ethical challenges (Evers, 2009; Lewis et al., 2014). First, PM must be accurate in order to not exclude individuals with ADHD from potentially effective care, and prioritize others arbitrarily, respectively. Second, stratification based on biomarkers may disadvantage those individuals who are excluded from the specified subgroups; see for example the discussion about use of polygenic risk scores (Martin et al., 2019). These might include ethnicities or minorities, who are already disadvantaged, which may increase stigmatization. Third, testing for a biomarker may also yield incidental findings that may either distract from or delay administration of the most effective treatment, and increase expense from unnecessary assessment, or cause more distress than relief to some patients. Fourth, biomarker studies also raise basic questions regarding access to health care, as private and public insurers could force individuals with ADHD first to conduct tests to determine coverage of therapies.

## A Roadmap Toward Personalized Medicine in ADHD

There are many tasks and challenges to negotiate in developing and implementing PM for ADHD. This needs to involve a variety of research designs and strategies, coupled with both hypothesis-free (for hypothesis generation) and hypothesis-driven (for hypothesis testing). Firstly, big data science, such as exploitation of large case-registry data bases, will be useful for exploratory analyses and hypothesis generation with regard to clinical and family-genetic markers of course and outcome. Infrastructure such as the United Kingdom Biobank also offers unprecedented opportunities for exploratory analyses capitalizing on access to genetic, brain imaging and other biological data and soon also to information on ADHD symptoms scores (Sudlow et al., 2015). Birth cohort or other population-based studies with a longitudinal design such as IMAGEN, ALSPAC and Generation-R provide additional opportunities for exploratory and discovery analyses on dimensional measures of ADHD (Golding, 2004; Schumann et al., 2010; Kooijman et al., 2016).

Secondly, the cornerstone will be to conduct prospective longitudinal cohort studies for the identification of valid, reliable, and clinically useful predictors, mediators, and moderators of clinical course, treatment response and long-term outcome across the age range. These will be observational within-subject studies of multi-site clinical ADHD cohorts that should involve careful documentation of treatments provided, assessment of clinical responses, and incorporation of potential biomarkers for diagnostic and/or stratification and/or prediction purposes. Outcomes studied might range from diagnostic accuracy to prediction of type of treatment, dosage needed, treatment response, various functional real-world outcomes, and course, but should also include cost-efficiency. There need to be discovery and validation cohorts, and studies across subgroups to take account of ethnic and cultural diversity, and allow for dissection of findings by age and sex.

Thirdly, incorporation of potential biomarkers in pharmaceutical industry-sponsored or investigator-initiated RCTs at baseline and eventually endpoint would be another powerful strategy to move forward. Special designs with enrichment strategies will improve statistical power for biomarker analyses (Freidlin and Korn, 2014). Further, as classical study designs are poor at addressing ADHD heterogeneity, future clinical studies using novel designs, e.g. stepped-care designs, are also required as to complement to the more standard efficacy RCTs (Lenze et al., 2020).

Fourthly, biomarker studies might benefit from a stronger orientation toward the dimensional perspective on ADHD and the Research Domain Criteria (RDoC) framework (Cuthbert, 2014). The goal of RDoC is to understand the nature of psychiatric disorders in terms of varying degrees of dysfunction in general psychological/biological systems or dimensions that cut across traditional diagnostic boundaries. The dimensions directly relevant for ADHD are, of course, the clinical domains of inattention and hyperactivity-impulsivity, but also executive functioning, reward processing, and arousal and regulation. An example is work on the overlap between ADHD and autism spectrum disorder (ASD) that showed that combined ADHD-ASD cases could be subtyped according to strong vs. weak visuo-spatial working memory (van der Meer et al., 2012, 2017).

An essential requirement is to establish rigorous standard operating procedures (SOPs) across sites for biomarker assessment, including achieving standards for test-retest reliability, across-site reproducibility and accuracy. Feasibility will also be an issue, as, for example, MRI biomarkers may be scientifically relevant as probes into the neural underpinnings of ADHD but less implementable into clinical practice. Appropriate statistical techniques for predictive modeling that may exploit the potential of advances machine learning approaches should be used (Wong et al., 2017; Fusar-Poli et al., 2018; Quaak et al., 2021). These can aid in the integration of complex multi-level data to achieve excellent predictive performance by taking into account the non-linear relationships and interactions that connect the set of predictors and the response variable (Fusar-Poli et al., 2018). Ecological momentary assessment devices allow, for example, collection of a huge amount of individualized real-time data within the natural environment (Perna et al., 2018). Probably, given the heterogeneity of ADHD, multiple biomarker panels may be more promising because the as yet unknown underlying pathophysiology may be reflected in different indices across individuals at multiple levels of assessment (behavior, cognition, brain structure and function, metabolomics, proteomics). For the latter, cutting-edge work on metabolomics and proteomics in ADHD is relatively absent and in need of upgrading. Biomarker research is an iterative process, where findings will improve our understanding of the underlying pathophysiology and conceptualization of ADHD. In turn, this will facilitate new studies on biomarkers discovery and validation.

Before implementation to routine clinical practice, rigorous evaluation of efficacy, and cost-effectiveness of PM diagnostic and treatment algorithms will be needed.

## Author Contributions

JB, TB, SC, LR, and SF developed the protocol for the search. AC and DL identified and retrieved the papers. TB, NC, and SC extracted the papers. JB and TB wrote the final version. All authors contributed to the writing of sections of the manuscript and contributed to the article and approved the submitted version.

## Funding

This work was supported by an unrestricted educational grant by Medice. BF is supported by a personal grant from the Innovation Program of the Netherlands Organization for Scientific Research (NWO; grant 016-130-669). GP is supported by São Paulo Research Foundation (FAPESP; grant 2016/22455-8) and National Council for Scientific and Technological Development (CNPq; grant 310582/2017-2). LR is supported by the National Council for Scientific and Technological Development (CNPq; grant 308294/2018-1). Medice was not involved in the study design, collection, analysis, interpretation of data, the writing of this article or the decision to submit it for publication.

## Conflict of Interest

JB has been in the past 3 years a consultant to/member of advisory board of / and/or speaker for Shire, Roche, Medice, and Servier. He is not an employee of any of these companies, and not a stock shareholder of any of these companies. He has no other financial or material support, including expert testimony, patents, royalties. LR has received grant or research support from, served as a consultant to, and served on the speakers' bureau of Eli Lilly and Co., Janssen, Medice, Novartis and Shire. The ADHD and Juvenile Bipolar Disorder Outpatient Programs chaired by LR have received unrestricted educational and research support from the following pharmaceutical companies: Eli Lilly and Co., Janssen, and Novartis. LR has received authorship royalties from Oxford Press and ArtMed and travel grants from Shire to take part in the 2018 APA annual meeting and from Novartis to take part of the 2016 AACAP annual meeting. Barbara Franke has received educational speaking fees from Medice and Shire. SB discloses that he has in the last 5 years acted as an author, consultant or lecturer for Shire, Medice, Roche, Eli Lilly, Prima Psychiatry, GLGroup, System Analytic, Ability Partner, Kompetento, Expo Medica, Clarion Healthcare, and Prophase. He receives royalties for text books and diagnostic tools from Huber/Hogrefe, Kohlhammer and UTB. DB serves as an unpaid scientific advisor for an EU-funded neurofeedback trial unrelated to the present work. TB served in an advisory or consultancy role for Actelion, Hexal Pharma, Lilly, Lundbeck, Medice, Neurim Pharmaceuticals, Novartis, Shire. He received conference support or speaker's fee by Lilly, Medice, Novartis and Shire. He has been involved in clinical trials conducted by Shire & Viforpharma. He received royalties from Hogrefe, Kohlhammer, CIP Medien, Oxford University Press. The present work is unrelated to the above grants and relationships. DC served in an advisory or consultancy role for Medice, Novartis, Shire/Takeda. He received conference support or speaker's fee by Medice, Servier and Shire/Takeda. He has been involved in clinical trials conducted by Shire. He received royalties from Oxford University Press. The present work is unrelated to the above grants and relationships. In the past year, SF received income, potential income, travel expenses continuing education support and/or research support from Tris, Otsuka, Arbor, Ironshore, Shire, Akili, Enzymotec, Sunovion, Supernus and Genomind. With his institution, he has US patent US20130217707 A1 for the use of sodium-hydrogen exchange inhibitors in the treatment of ADHD. In previous years, he received support from: Shire, Ironshore, Neurovance, Alcobra, Rhodes, CogCubed, KemPharm, Enzymotec, Akili, Neurolifesciences, Lundbeck/Takeda, Otsuka, McNeil, Janssen, Novartis, Pfizer and Eli Lilly. He also receives royalties from books published by Guilford Press: Straight Talk about Your Child's Mental Health, Oxford University Press: Schizophrenia: The Facts and Elsevier: ADHD: Non-Pharmacologic Interventions. He is principal investigator of www.adhdinadults.com. Guilherme V. Polanczyk has been in the past 3 years a consultant, member of advisory board, and/or speaker for Shire/Takeda and Medice. He received travel expenses for continuing education support from Shire/Takeda and royalties from Editora Manole. In the past three years, Jeffrey Newcorn is/has been an advisor and/or consultant for Adlon Therapeutics, Akili Interactive, Alcobra, Arbor, Cingulate Therapeutics, Enzymotec, KemPharm, Lundbeck/Takeda, Medice, NLS, Rhodes, Shire, and Supernus. He was a DSMB member for Pfizer and Sunovion, and received research funds from Enzymotec, Otsuka, Shire and Supernus. He also has received speaker fees from Shire for disease-state presentations, and served as a consultant for the US National Football League. BV has served as consultant to Medice and Teva Pharm. Markus Gleitz is an employee of Medice Arzneimittel Pütter GmbH & Co. KG. MS received research support from Supernus, Shire, and Akili Interactive labs and in the past year has served in a advisory or consultancy capacity to Genomind, NLS, Shire/Takeda, Supernus, and Sunovian. The remaining authors declare that the research was conducted in the absence of any commercial or financial relationships that could be construed as a potential conflict of interest. The reviewer KAJ declared a shared affiliation with the author DC to the handling editor at the time of review.

## Publisher's Note

All claims expressed in this article are solely those of the authors and do not necessarily represent those of their affiliated organizations, or those of the publisher, the editors and the reviewers. Any product that may be evaluated in this article, or claim that may be made by its manufacturer, is not guaranteed or endorsed by the publisher.
