# Supplementary material for: Toward Precision Medicine in ADHD
Source: Front Behav Neurosci. 2022 Jul 6;16:900981. doi: 10.3389/fnbeh.2022.900981 (PMC9299434; doi:10.3389/fnbeh.2022.900981)
Supplement: Supplementary file 1 [file Presentation_1.pdf]

Supplementary Materials

Precision Medicine in ADHD: protocol for a systematic review

Jan Buitelaar, Samuele Cortese, Tobias Banaschewski, Luis Rohde, Stephen Faraone

Background

Attention-Deficit/Hyperactivity Disorder (ADHD) is a complex and heterogeneous neurodevelopmental condition for which curative treatments are lacking [1]. Whilst pharmacological treatments are generally effective and safe [2], there is considerable inter-individual variability among patients regarding treatment response, required dose, and tolerability. Many of the non-pharmacological treatments, which are preferred to drug-treatment by some patients, either lack efficacy for core symptoms or are associated with small effect sizes [3]. No evidence-based decision tools are currently available to allocate pharmacological or psychosocial treatments based on the patient's clinical, environmental, cognitive, genetic, or biological characteristics.
The success of precision medicine (PM) depends on identifying biomarkers, especially stratification biomarkers. A biomarker measures the presence or severity of a condition, its course or treatment response; it can be a physical, chemical, biologic, genetic, or psychological measure. Unfortunately, many attempts to identify, validate and implement biomarkers in psychiatry have failed, for example because of lack of (or adherence to) rigorous standardized operating procedures, small sample sizes and failures of replication. To be implementable in the clinic, a potential biomarker should be validated across independent data sets, and be reproducible, reliable, specific, sensitive, clinically relevant and feasible. To our knowledge, a comprehensive and updated synthesis of the literature on biomarkers for a precision medicine approach for ADHD is lacking.

Review questions

The overarching goal of the systematic review is to establish, through the available literature, candidate biomarkers to be used in precision medicine approaches to ADHD.

More in particular, this is the protocol of a systematic review of randomised controlled trials (RCTs|) or non randomised studies exploring cognitive, physiological (EEG/ERP), MRI, genetic and clinical measures that may serve as biomarkers to stratify ADHD into more homogenous subgroups and/or predict response to treatment and/or predict course.

The main review question is: which are the measures that have been shown to predict diagnosis, response and/or tolerability to treatment, or clinical course in ADHD?

Search strategy/syntax

We will search Pubmed, PsycInfo, EMBASE+EMBASE classic, OVID Medline and Web of Science databases (WEB OF SCIENCE databases (Web of Science Core Collection, Biological Abstracts, BIOSIS Citation Index, Current Contents Connect, Data Citation Index, Derwent, Innovations Index, FSTA® - the food science resource, KCI-Korean Journal Database MEDLINE®, Russian Science Citation Index, SciELO Citation Index, Zoological

Record) from 1.1.2000, with no language and type of document restrictions. The syntax and search terms for each database are as follows:

PUBMED

- Search terms:

((ADHD [tiab] OR attention deficit [tiab] OR attention-deficit [tiab] OR hyperkinetic syndrome [tiab] OR hyperkinetic disorder [tiab]) AND (Marker [tiab] OR biomarker [tiab] OR diagnostic marker [tiab] OR stratification marker [tiab] OR predictive marker [tiab] OR prognostic marker [tiab] OR prediction response [tiab] OR prediction course [tiab] OR precision medicine [tiab] OR personalized medicine [tiab] OR positive predictive value [tiab] OR negative predictive value [tiab] OR positive predictive power [tiab] OR negative predictive power [tiab] OR sensitivity [tiab] OR specificity [tiab] OR area under the curve [tiab] OR AUC [tiab]) AND (random* [tiab] OR clinical trial [tiab] OR crossover [tiab] OR cross-over [tiab] OR case-control [tiab] OR cohort study [tiab] or cross-sectional [tiab] OR longitudinal [tiab] OR prospective [tiab])) NOT screen* [tiab]

OVID databases

PsycInfo, EMBASE+EMBASE classic, OVID Medline

Search terms:

((ADHD OR attention deficit OR attention-deficit OR hyperkinetic syndrome OR hyperkinetic disorder).ti,ab AND (Marker OR biomarker OR diagnostic marker OR stratification marker OR predictive marker OR prognostic marker OR prediction response OR prediction course OR precision medicine OR personalized medicine OR positive predictive value OR negative predictive value OR positive predictive power OR negative predictive power OR sensitivity OR specificity OR area under the curve OR AUC).ti,ab AND (randomised or randomized Or random OR clinical trial OR crossover OR cross-over OR case-control OR cohort study or cross-sectional OR longitudinal OR prospective).ti,ab) NOT (screening OR screen).ti,ab

WEB OF SCIENCE databases (Web of Science Core Collection, Biological Abstracts, BIOSIS Citation Index, Current Contents Connect, Data Citation Index, Derwent, Innovations Index, FSTA® - the food science resource, KCI-Korean Journal Database MEDLINE®, Russian Science Citation Index, SciELO Citation Index, Zoological Record)

Search terms:

(ADHD OR attention deficit OR attention-deficit OR hyperkinetic syndrome OR hyperkinetic disorder

AND

Marker OR biomarker OR diagnostic marker OR stratification marker OR predictive marker OR prognostic marker OR prediction response OR prediction course OR precision medicine OR personalized medicine OR positive predictive value OR negative predictive value OR positive predictive power OR negative predictive power OR sensitivity OR specificity OR area under the curve OR AUC

AND

randomised or randomized Or random OR clinical trial OR crossover OR cross-over OR case-control OR cohort study or cross-sectional OR longitudinal OR prospective

NOT (screen*)

References list of relevant will be hand-searched to detect any relevant reference missed during the electronic search. Additionally, experts in the field will be contacted to query about any additonal relevant reference.

Types of studies to be included

We will include randomised controlled studies (parallel or cross-over) and observational studies (case-control, cohort, cross-sectional). For prospective studies, we will extract data at baseline or at the earliest time point.

Participants/population

ADHD: The population of interest will include children and/or adults with either:
1) a categorical diagnosis of ADHD according to the DSM (III, III-R, IV, IV-TR or 5) or Hyperkinetic Disorder (HD) as per the ICD-10 or previous ICD versions; or
2) a definition of ADHD using a symptoms threshold on a validated ADHD rating scales; or
3) for adults, a positive answer to the question: “Did your doctor ever tell you that you have ADHD?”); or
4) a diagnosis of ADHD recorded in medical files/registries.
We will exclude studies assessing only symptoms of ADHD, without a diagnosis. We will also exclude studies including participants with a diagnosis of Minimal Brain Dysfunction (MBD), which would not be comparable with DSM definitions of ADHD, or with DAMP syndrome (Deficit in Attention, Motor control and Perception), since this category is controversial.
Age and gender: Studies including individuals of any age and of both genders will be retained.

Outcomes

Relationship between (bio)markers and prediction of diagnosis, response to treatment/tolerability, or prediction of course of ADHD

Data extraction (selection and coding)

Studies identified with electronic and manual searches will be listed with citation, titles and abstracts from all databases in Endnote (Clarivate ®); duplicates will be excluded using the Endnote function “remove duplicates”

The eligibility process will be conducted in two separate stages:

1. Two authors will independently screen title and abstracts of all non duplicated papers and will exclude those not pertinent. A final list will be agreed with discrepancies resolved by consensus between the two authors. When consensus is not reached, a third, senior author will act as arbitrator. If any doubt about inclusion exists, the article will proceed to the next stage.
2. The full-text version of the articles passing stage 1 screening will be downloaded and assessed for eligibility by two authors, independently. Discrepancies will be resolved by consensus between the two authors and, if needed, a third senior author will act as arbitrator.

Where required, we will contact the corresponding author to inquire on study eligibility.

Data extraction

Two researchers will perform independently data extraction; any discrepancies will be resolved by consensus between the two authors. If this is not possible, another senior author will make a judgement on the data entered and act as an arbitrator.

Data will be extracted and inserted in an Excel sheet. The following data will be extracted:

1. Design: type of study (cross-sectional, case-control, cohort, etc.); study temporality (prospective, retrospective); patient enrolment (consecutive, non-consecutive); setting (clinical vs. population-based study);
2. Study participants details: number, mean age (SD), gender distribution, SES and ethnicity of participants with and without ADHD; characteristics of participants without ADHD (healthy comparisons, comparisons with psychiatric disorders other than ADHD, other); psychiatric comorbidities of individuals with and without ADHD (type and prevalence); method to establish the diagnosis of ADHD (self-reported diagnosis, diagnosis recorded in medical files/registry, structured or semi-structured interview according to DSM (III, III-R, IV. IV-TR, 5) or ICD (ICD-10 or previous versions) criteria); medication status of individuals with and without ADHD (type of medication and percentage of treated participants, during and prior to the study);
3. Outcome: Diagnosis of ADHD or response to treatment or course of ADHD.

Additionally the following aspects will be included in the data extraction:

- clinical, comorbidity, gender/sex, age, severity of symptoms
- EEG/ERP, neurophysiology, psychophysiology
- MRI, neuroimaging
- Cognition, neurocognition, neuropsychology, attention, inhibition, executive function, memory

- Levels, peripheral, serum, plasma, urine, saliva, blood, platelets, cerebrospinal fluid, red blood cells, hair in combination with: dopaminergic, noradrenergic, serotonergic, biogenic trace amines systems, and their principal metabolites; environmental risk factors, including heavy metals and substance/chemical exposures and nutritional factors; hypothalamic–pituitary–adrenal axis (HPA) alterations; and markers involved in other aspects of brain functioning (growth hormone and thyroid function, oxidative stress cascade, cytokine unbalance, other neurotransmission systems, neurotrophic factors, complement C4-B, pineal hormone melatonin.

Strategy for data synthesis
N/A (systematic review)

Analysis of subgroups or subsets
N/A (systematic review)

References

1. Faraone SV, Asherson P, Banaschewski T, et al. Attention-deficit/hyperactivity disorder. Nat Rev Dis Primers 2015;1:15020.
2. Cortese S, Adamo N, Del Giovane C, et al. Comparative efficacy and tolerability of medications for attention-deficit hyperactivity disorder in children, adolescents, and adults: a systematic review and network meta-analysis. Lancet Psychiatry 2018;5:727-738.
3. Sonuga-Barke EJ, Brandeis D, Cortese S, Daley D, Ferrin M, Holtmann M, Stevenson J, Danckaerts M, van der Oord S, Dopfner M, Dittmann RW, Simonoff E, Zuddas A, Banaschewski T, Buitelaar J, Coghill D, Hollis C, Konofal E, Lecendreux M, Wong IC, Sergeant J. Nonpharmacological interventions for ADHD: systematic review and meta-analyses of randomized controlled trials of dietary and psychological treatments. *Am J Psychiatry.* 2013;170(3):275-289.
